# Impact of alcohol exposure on neural development and network formation in human cortical organoids

**DOI:** 10.1038/s41380-022-01862-7

**Published:** 2022-11-16

**Authors:** Jason W. Adams, Priscilla D. Negraes, Justin Truong, Timothy Tran, Ryan A. Szeto, Bruno S. Guerra, Roberto H. Herai, Carmen Teodorof-Diedrich, Stephen A. Spector, Miguel Del Campo, Kenneth L. Jones, Alysson R. Muotri, Cleber A. Trujillo

**Affiliations:** 1 Department of Pediatrics/Rady Children’s Hospital, Department of Cellular & Molecular Medicine, University of California San Diego, School of Medicine, La Jolla, CA 92037 USA; 2 Department of Neurosciences, University of California San Diego, School of Medicine, La Jolla, CA 92093 USA; 3 Center for Academic Research and Training in Anthropogeny, University of California San Diego, La Jolla, CA 92093 USA; 4 Experimental Multiuser Laboratory, Pontifícia Universidade Católica do Paraná, Curitiba, PR 80215-901 Brazil; 5 Department of Pediatrics, Division of Infectious Diseases, University of California San Diego, La Jolla, CA 92093 USA; 6 Department of Pediatrics, Division of Dysmorphology and Teratology, University of California, La Jolla, CA 92093 USA

**Keywords:** Stem cells, Neuroscience

## Abstract

Prenatal alcohol exposure is the foremost preventable etiology of intellectual disability and leads to a collection of diagnoses known as Fetal Alcohol Spectrum Disorders (FASD). Alcohol (EtOH) impacts diverse neural cell types and activity, but the precise functional pathophysiological effects on the human fetal cerebral cortex are unclear. Here, we used human cortical organoids to study the effects of EtOH on neurogenesis and validated our findings in primary human fetal neurons. EtOH exposure produced temporally dependent cellular effects on proliferation, cell cycle, and apoptosis. In addition, we identified EtOH-induced alterations in post-translational histone modifications and chromatin accessibility, leading to impairment of cAMP and calcium signaling, glutamatergic synaptic development, and astrocytic function. Proteomic spatial profiling of cortical organoids showed region-specific, EtOH-induced alterations linked to changes in cytoskeleton, gliogenesis, and impaired synaptogenesis. Finally, multi-electrode array electrophysiology recordings confirmed the deleterious impact of EtOH on neural network formation and activity in cortical organoids, which was validated in primary human fetal tissues. Our findings demonstrate progress in defining the human molecular and cellular phenotypic signatures of prenatal alcohol exposure on functional neurodevelopment, increasing our knowledge for potential therapeutic interventions targeting FASD symptoms.

## Introduction

Despite public health efforts, prenatal alcohol exposure (PAE) remains the leading preventable cause of neurodevelopmental disorders [1]. Alcohol (EtOH) exposure during pregnancy can result in Fetal Alcohol Spectrum Disorders (FASD) [2–4], which together have a prevalence of approximately 2–5% in the United States alone [4, 5]. FASD can present with a wide range of symptomatic severity, from neurobehavioral abnormalities to embryonic lethality. Primary cognitive and behavioral deficits can underpin secondary disabilities, such as mental health problems, disrupted school experience, addiction, sexually deviant behavior, and dependent living [3, 6–9]. The resultant clinical profile appears to be influenced by the fetal brain’s stage of development at the time of exposure and pattern of maternal EtOH consumption [10–16].

Although the public burden of FASD is recognized, the precise molecular pathophysiology and neurodevelopmental consequences of EtOH exposure in utero remain understudied in humans. Previous investigations using in vitro and animal models have identified a broad impact of EtOH on neural functioning, with effects dependent upon cell type and the severity of exposure [9–16]. However, translating these findings to clinical application in humans has been challenged by the suboptimal recapitulation of human cortical development by animal models [17]. Human induced pluripotent stem cells (hiPSCs) provide an experimental model that supports interrogation of the pathophysiological effects of EtOH exposure in a dynamic neurodevelopmental context [18]. Most valuably, hiPSCs can be aggregated and differentiated into three-dimensional cortical organoids that develop closely to human fetal corticogenesis and can be used to study mechanisms of neurodevelopmental disease [19–27].

Here, we identified EtOH-induced, temporally specific alterations at neurodevelopmental time points in hiPSC-derived cortical organoids and astrocytes, and we validated these findings in primary human fetal tissue. EtOH exposure alters the accessibility of the chromatin regulatory landscape in a pattern that is proposed to overlie transcriptomic alterations, cellular phenotypic changes, and impairment of functional network formation. Our findings suggest that this cortex-specific neurodevelopmental model can be a valuable platform to study the vulnerability of the early human brain to environmental damage and offers urgently needed insights into the pathophysiology of PAE.

## Materials and methods

### Human cell source (hiPSC and primary fetal tissues)

Three hiPSC lines, WT83C6 (male), CVB (male), and 4C1 (female), were derived from control individuals with informed consent and have been previously characterized elsewhere [28–30]. The hiPSC colonies were expanded on Matrigel-coated dishes (BD Biosciences, San Jose, CA, USA) with mTeSR1 medium (StemCell Technologies, Vancouver, Canada). The cells were routinely checked by karyotype and CNV arrays to avoid genomic alterations in the culture. Embryonic samples were obtained from fetal brains (10–11 weeks post-conception (PCW)) and cultured in Neurobasal (Life Technologies, Carlsbad, CA, USA) supplemented with 1X GlutaMAX (Life Technologies), 1% Gem21 NeuroPlex (Gemini Bio-Products, West Sacramento, CA, USA), 1% MEM nonessential amino acids (NEAA; Life Technologies), and 1% penicillin/streptomycin (Pen/Strep; Life Technologies). All cellular cultures were routinely tested for mycoplasma by PCR. The study was approved by the University of California San Diego IRB/ESCRO committee (protocol 141223ZF).

### Alcohol (EtOH) exposure strategy

Cortical organoids were exposed to EtOH by supplementing the culture media with 200-proof EtOH (100 mM, Sigma–Aldrich) for seven days, with media changes every other day. After this period, cells were maintained in media without EtOH for as long as needed. The same EtOH exposure approach was performed in mature primary fetal neurons and hiPSC-derived astrocytes. Although cortical organoids, fetal neurons, and astrocytes were initially exposed to 100 mM EtOH, the final EtOH concentration in the media stabilized around 20 mM after a couple of hours, as demonstrated by over-time measurements using the DensitoPro Handheld Density Meter (Mettler Toledo, Columbus, OH, USA). Cells were incubated at 37 °C throughout treatment. Control and treated cell types batches were kept separately in distinct and covered conditions with no cross-contamination or EtOH leakage. In order to maintain a stable environmental concentration close to 20 mM and avoid evaporation of EtOH, cells were always kept in an EtOH-saturated atmosphere.

### Epigenetic profile (assay for transposase accessible chromatin sequencing; ATAC-Seq)

Samples were cryopreserved in a solution containing 50% FBS/40% maintenance media/10% DMSO and transferred to Active Motif (Carlsbad, CA, USA), where the assay was performed according to their optimized protocol. Data was analyzed by Rosalind (https://rosalind.onramp.bio/) using a HyperScale architecture developed by OnRamp BioInformatics, Inc. (San Diego, CA, USA). Peaks were called using MACS2 [31]. Peak overlaps and differential accessibility were calculated using the DiffBind R library. Differential accessibility was calculated at gene promoter sites. Read distribution percentages, identity heatmaps, and FRiP plots were generated as part of the QC step using ChIPQC R library and HOMER [32]. HOMER was also used to generate known and *de novo* motifs and perform functional enrichment analysis of pathways, gene ontology, domain structure, and other ontologies.

### Histone extraction and mass spectrometry

Bulk histones were acid-extracted from cell pellets, propionylated, and subjected to trypsin digestion as previously described [33]. Briefly, histones were extracted by incubation and intermittent vortexing. Histones were then precipitated, recovered, washed twice with PBS, and air-dried. While adjusting the pH to 7–8, the pelleted histones were resuspended and digested with trypsin before resuspension in 100 mM ammonium bicarbonate overnight at 37 °C and dried again. For mass spectrometry analysis, histone peptides were resuspended in 0.1% TFA in water.

Samples were analyzed in a triple quadrupole (QqQ) mass spectrometer (TSQ Quantiva, Thermo Fisher Scientific) directly coupled with an UltiMate 3000 nano-liquid chromatography system (Dionex, Sunnyvale, CA, USA). Peptides were first loaded onto a packed trapping column and then separated on a New Objectives PicoChip analytical column. Both columns were packed with New Objectives ProntoSIL C18-AQ, 3 μm, 200 Å resin. The chromatography gradient was achieved by increasing the percentage of buffer B from 0 to 35% at a flow rate of 0.30 μL/min over 45 min. Solvent A: 0.1% formic acid in the water, and B: 0.1% formic acid in 95% acetonitrile. The QqQ settings were as follows: collision gas pressure of 1.5mTorr; Q1 peak width of 0.7 (FWHM); cycle time of 2 s; skimmer offset of 10 V; electrospray voltage of 2.5 kV. In addition, targeted analysis of unmodified and various modified histone peptides was performed. This entire process was repeated three separate times for each sample.

Raw MS files were imported and analyzed in Skyline with Savitzky-Golay smoothing [34]. All Skyline peak area assignments for monitored peptide transitions were manually confirmed. A minimum of three peptide transitions was quantified for each modification. For each monitored amino acid residue, each modified (and unmodified) form was quantified by first calculating the sum of peak areas of corresponding peptide transitions. Finally, each modification is represented as a percentage of the total pool of modifications. In other words, for each individual modification (including unmodified), the data for each sample is converted to the fraction of the sum across all samples to display the relative abundance of particular modifications across the sample group.

### Cell cycle analysis

Cortical organoids, primary fetal neurons, and astrocytes were manually dissociated and counted using a Via1-Cassette with the NucleoCounter NC-3000 (Chemometec, Allerod, Denmark). Dissociated cells were fixed with 70% EtOH for two hours, resuspended in 0.5 µg/ml DAPI with 0.1% Triton X-100 in PBS, and incubated at 37 °C for five minutes. Cells were subsequently loaded into an NC‐Slide A2 chamber (Chemometec), and cellular fluorescence was quantified with the NucleoCounter NC-3000 using the manufacturer’s protocol.

### Annexin and cell death

Cortical organoids, primary fetal neurons, and astrocytes were manually dissociated and resuspended in Annexin V binding buffer (Invitrogen). Next, Annexin V-CF488A conjugate (Biotium, Inc., Hayward, CA, USA) was added, followed by Hoechst 33342 (Chemometec). After a PBS wash, the cells were resuspended in Annexin V binding buffer (Invitrogen) containing 10 μg/mL propidium iodide (Chemometec). Cells were loaded into NC‐Slide A2 chambers, and the “Annexin V Assay” was run with the NucleoCounter NC-3000.

### Synaptic puncta quantification

Pre (Vglut1+) and postsynaptic (PSD95+) puncta were quantified along MAP2+ neuronal processes, as previously described [29]. The primary antibodies (Vglut1, 1:1000, Synaptic Systems; PSD95, 1:1000, NeuroMab; and MAP-2, 1:2000, Sigma–Aldrich) were incubated for two hours at room temperature. After wash, secondary antibodies (Alexa Fluor 488-, 555- and 647-conjugated antibodies, 1:1000, Life Technologies) were incubated for one hour. Coverslips were mounted, and slides were analyzed under a fluorescence microscope (Z1 Axio Observer Apotome, Zeiss).

### Nanostring technologies GeoMx digital spatial profiling

The Nanostring Technologies GeoMx Digital Spatial Profiling (DSP) platform quantifies protein abundance by counting indexing oligonucleotides specific to the desired targets [35]. EtOH-exposed and control organoid sections were stained with fluorescently labeled imaging reagents and select protein markers as described [35]. Briefly, sections were labeled with the cell markers Ki-67, GFAP, MAP2, and nuclear stain. The Nanostring commerical protein panels Neural Cell Profiling, Alzheimer’s Pathology, and Glial Cell Subtyping were selected for biological target proteins. Slides were scanned on a GeoMx DSP instrument, and specific regions or areas of interest (ROI or AOI) were chosen for profiling [35]. Regions selected were rosettes and non-rosettes containing areas within organoids from each condition. UV-cleaved indexing oligonucleotides were collected and reads were digitally quantified on a Nanostring nCounter, and analyses were performed using GeoMx DSP Data Analysis software [35].

### Drug treatment strategy

Donepezil (Tocris, Minneapolis, MN, USA), Nefiracetam (Tocris), and IGF-1 (Peprotech) were prepared according to manufacturer specifications. EtOH-exposed organoids that reached 2.5 months of age began 3 weeks of treatment with drug compounds, with each drug supplemented at 1 μM in regular media changes 3x/week. Corresponding vehicle volumes of DMSO or deionized water were administered to EtOH-exposed controls. Organoids were collected and assayed immediately following the drug treatment window.

### Multi-electrode array (MEA) recording

MEA recordings were achieved as previously described [23, 30]. Six-week-old cortical organoids were plated in 12-well MEA plates (Axion Biosystems, Atlanta, GA, USA) pre-coated with 100 µg/mL poly-L-ornithine and 10 µg/mL laminin. Cellular cultures were fed twice a week and, 14 days after plating, weekly measurements were performed. Recordings were conducted in a Maestro MEA system with AxIS Software Spontaneous Neural Configuration (Axion Biosystems). Using Axion Biosystems’ Neural Metrics Tool, active electrodes required at least 5 spikes/min. Bursts/electrode used an inter-spike interval (ISI) threshold requiring minimally 5 spikes with a maximum ISI of 100 ms. Network bursts required at least 10 spikes under the same ISI. The synchrony index utilized a cross-correlogram window of 20 ms.

### Statistical analysis

Statistical analysis was performed using GraphPad Prism versions 8 and 9 (GraphPad Software, La Jolla, CA, USA). Sample sizes were mainly determined based on our previous experiments and previous lab publications, although the sample size for drug rescue experiments was limited by time availability; no formal randomization was used to allocate samples to experimental condition. Results from continuous variables are presented as mean ± standard error of the mean (s.e.m.), and 95% confidence intervals were normal-based. Means were compared between groups using, where appropriate, unpaired Student’s *t*-test, one-way, or two-way analyses of variance (ANOVA). Outliers were determined using GraphPad criteria. Whenever possible, the investigator was blind to the sample conditions. Tests were performed two-sided with α throughout set as 0.05.

## Results

### Human cellular models to study the neurodevelopmental effects of EtOH exposure

**Fig. 1:**
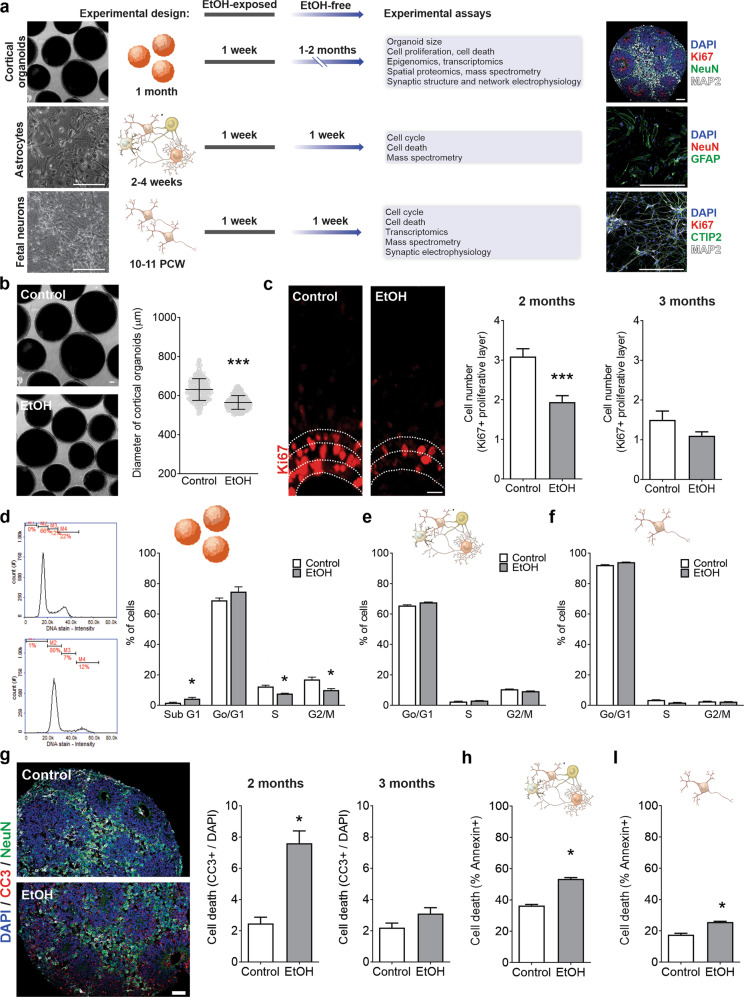
Ethanol (EtOH) exposure alters proliferation and survival dynamics in human cellular models. **a** EtOH was applied to ~4-week-old human iPSC-derived cortical organoids, ~2–4-week-old human iPSC-derived astrocytes, and human 10–11 post-conception weeks (PCW) fetal primary neurons. **b** Cortical organoid diameter is decreased by EtOH exposure (Student’s *t*-test, *t*_541_ = 17.05, *P* < 0.0001; *n* = 210–333 organoids/condition). **c** Immunohistochemistry portrays layers of Ki67+ proliferation (two months, Student’s *t*-test, *t*_64_ = 4.518, *P* < 0.0001; three months, Student’s *t*-test, *t*_18_ = 1.63, *P* = 0.12; *n* = 10–33 organoids/condition using WT83, CVB, and 4C1 cell lines). **d**–**f** EtOH exposure produces cell cycle alterations in cortical organoids (two-way analysis of variance (ANOVA), *F*_3,16_ = 8.14, *P* = 0.002; *n* = 3 replicates/conditio*n*, from WT83, CVB, and 4C1 cell lines) (**d**), iPSC-derived astrocytes (two-way ANOVA, *F*_2,42_ = 17.06, *P* < 0.0001; *n* = 8 replicates/condition, from WT83 and 4C1 cell lines) (**e**), and fetal neurons (two-way ANOVA, *F*_2,42_ = 41.92, *P* < 0.0001; *n* = 8 replicates/condition) (**f**). **g**–**i** EtOH exposure promotes cell death, shown by CC3+ immunohistochemistry in cortical organoids (two months, Student’s *t*-test, *t*_28_ = 5.785, *P* < 0.0001; three months, Studen*t*’s *t*-test, *t*_18_ = 1.886, *P* = 0.076; *n* = 10–15 organoids/condition from WT83 cell line) (**g**) a*n*d Annexin+ in astrocytes (Student’s *t*-test, *t*_14_ = 15.03, *P* < 0.0001; *n* = 8 replicates/condition from WT83 and 4C1 cell lines) (**h**) and fetal neurons (Student’s *t*-test, *t*_6_ = 7.872, *P* = 0.0002; *n* = 4 replicates/condition) (**i**). Data are presented as mean ± standard error of the mean (s.e.m.). Scale bar = 100 µm.

To investigate the neurodevelopmental consequences of EtOH exposure, human cell platforms encompassing diverse neural cell types were employed. We derived functional cortical organoids and astrocytes from three hiPSC lines (Fig. 1a and Supplementary Fig. 1b–d, e) and prepared primary neuron cultures from human fetal tissue at 10-11 PCW (Fig. 1a and Supplementary Fig. 1h). Each cell type underwent moderate-high physiological concentration of EtOH exposure for one week (~4-week-old organoids, fetal neurons, and ~2–4-week-old astrocytes), followed by continued maintenance—fetal neurons and astrocytes for one week and one-to-two months for cortical organoids—in the absence of EtOH, at the end of which experimental assays were performed (Fig. 1a). Although the choice to administer 100 mM of EtOH is high, the concentration to which the cells were kept corresponds to around 20 mM or 90 mg/dL (Supplementary Fig. 1a). This treatment dosage was selected considering EtOH cytotoxicity and its concentration in amniotic fluid [36–39].

Immunocytochemistry and transcriptomics indicated close fidelity between cortical organoids and human fetal cortical development. Immunocytochemistry portrayed SOX2+ progenitor regions enriched for proliferation (Ki67+) organized in ventricular zone (VZ)-like areas around a lumen, giving rise to a cortical plate-like area displaying mature neurons (NeuN+, MAP2+) that express the cortical laminar-specific markers CTIP2 and SATB2 (Supplementary Fig. 1b, c). Furthermore, transcriptomic comparison of two- and three-month-old organoids (see below) with the BrainSpan human brain gene expression reference [40] showed close correlation between organoids and developing 9–13 PCW fetal cortex (Supplementary Fig. 1d and Supplementary Table 1). Because glia cells emerge later during neurodevelopment and are thus absent or sparse in early cortical organoids [23] (Supplementary Fig. 1c), we differentiated astrocytes independently to capture astrocytic alteration attributable to EtOH exposure.

### EtOH exposure alters cell cycle, proliferation, and survival

EtOH-exposed cortical organoids showed reduced diameter at two months compared to controls (*P* < 0.0001; Fig. 1b). Ki67+ staining was used to delineate the proliferative VZ-like region from the cortical plate-like domain, as previously described in a cortical organoid system [41]. At two months of age, a stage in organoid development with considerable cell proliferation, we observed a smaller population of Ki67+ proliferative cells in EtOH-exposed cultures compared to controls (*P* < 0.0001); however, at three months, this difference had narrowed (*P* = 0.12; Fig. 1c). The overall amount of cells (DAPI+) remained constant, independent of treatment. Next, cell cycle analysis showed that cells from EtOH-exposed organoids were less likely to be in the proliferative S and G2/M phases (*P* = 0.002; Fig. 1d). Similar alterations in cell cycle were also observed in EtOH-exposed astrocytes (*P* < 0.0001; Fig. 1e and Supplementary Fig. 1g) and fetal neurons (*P* < 0.0001; Fig. 1f and Supplementary Fig. 1j).

After identifying alterations in proliferation and cell cycle progression, we investigated the effect of EtOH exposure on cell survival and found a higher frequency of apoptotic cells (CC3+) in two-month EtOH-exposed organoids compared to controls (*P* < 0.0001; Fig. 1g). Analogous to the proliferative impairment, we observed that by three months the increased frequency of apoptosis in EtOH-exposed organoids had comparatively stabilized (*P* = 0.076; Fig. 1g). To evaluate cell death in astrocytes and fetal neurons, we assessed the frequency of Annexin+ cells, which was also increased in both astrocytes (*P* < 0.0001; Fig. 1h and Supplementary Fig. 1f) and fetal neurons (*P* = 0.0002; Fig. 1i and Supplementary Fig. 1i) exposed to EtOH.

### Epigenetic landscape during neurodevelopment is altered by EtOH exposure

**Fig. 2:**
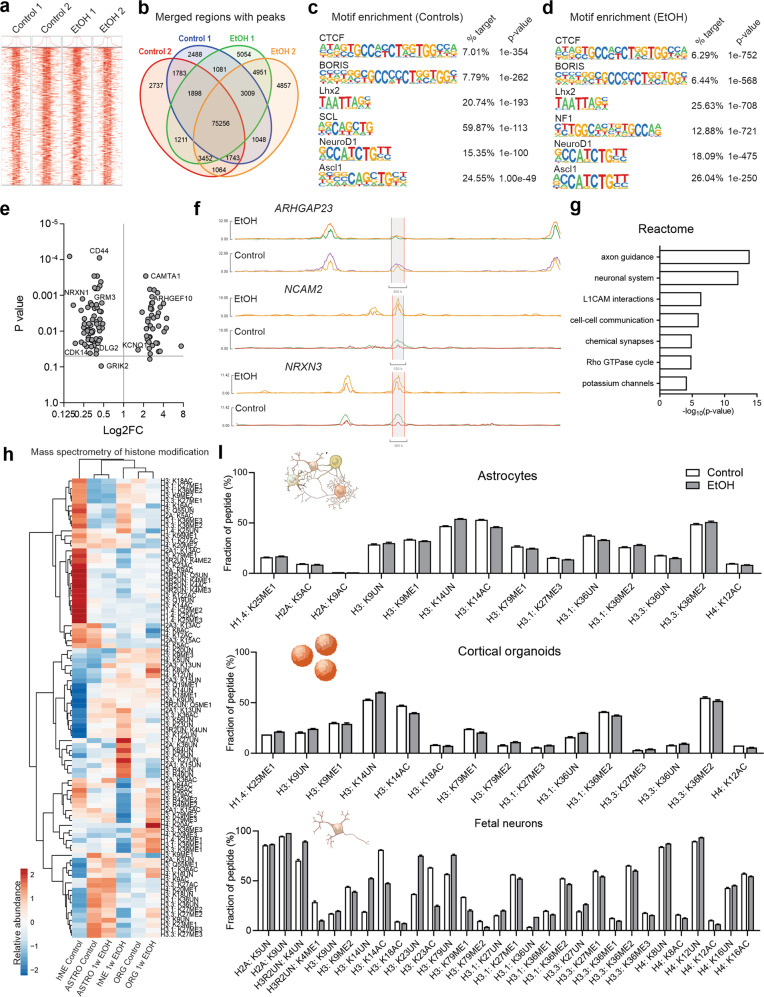
EtOH affects chromatin accessibility in regions critical for neurodevelopment. **a**–**g** ATAC-seq analysis of two-month old control and EtOH-exposed cortical organoids. **a** Transcription start site (TSS) enrichment plot showing TSS ± 1.0 Kb for each sample. ‘Control 1’, ‘Control 2’, ‘EtOH 1’, and ‘EtOH 2’ labels denote independent batches of organoids made from the WT83 cell line. **b** Venn diagram depicts peaks in EtOH-exposed and control organoids. **c**, **d** Motif enrichment in control (**c**) and EtOH-exposed (**d**) organoids. **e** Plotting enrichment *P* values identifies prominent regions of altered accessibility. **f** Tracks plot of select prominent genes. **g** Reactome analysis predicts that the effects of altered chromatin accessibility concentrate in physiological processes central to neurodevelopment. **h**, **i** Mass spectrometry analysis shows histone modifications in astrocytes, cortical organoids, and fetal neurons (hNE) attributable to EtOH-exposure; cortical organoids and astrocytes were generated from the WT83 iPS cell line. Heatmap shows EtOH exposure modifies histone methylation (ME) and acetylation (AC) patterns in diverse neural cell types; scale bar portrays relative abundance of modification across samples: red=high compared to other samples, blue=low compared to other samples (**h**). A recurrent pattern of modifications is observed between astrocytes, organoids, and fetal neurons; only statistically significant differences in relative modification abundance are shown (**i**).

Epigenetic signaling is the principal mediator of the transcriptional response to environmental stimuli, a dynamic process in which stimulus-induced shifts in chromatin accessibility modulate differential gene expression. To define alterations in the chromatin human neurodevelopmental regulatory landscape due to EtOH exposure, we performed ATAC-seq in control and EtOH-exposed cortical organoids [42]. A high-quality library (Q30 > 90) was generated for each sample, and we detected a total of 604,998 peaks (Supplementary Tables 2 and 3); EtOH-exposed organoids exhibited 20,731 peaks that were both common between replicates and distinct from controls. Annotating the peaks by genomic region suggested preferential enrichment of intergenic regions expected to have a regulatory function (Fig. 2a, b, and Supplementary Fig. 2a–e). Analysis of chromatin motifs showed an association of EtOH exposure with enrichment of different sequences linked with proliferation and neurogenesis, including *NF1*, *NeuroD1*, and *Ascl1* (Fig. 2c, d). For instance, *ARHGEF10* and *ARHGAP23* are linked with rho GTPases, and *NRXN1* and *NRXN3* produce synaptic cell adhesion molecules to promote synaptic structural integrity (Fig. 2e, f). A summary Reactome analysis confirmed the prominent neurodevelopmental consequences of EtOH, with affected functions including axon guidance, cellular adhesion, and synaptic communication (Fig. 2g).

In order to extend our ATAC-seq findings, we performed mass spectrometry to characterize EtOH-induced histone modifications in astrocytes, cortical organoids, and primary fetal neurons. Compared to controls, EtOH exposure was associated with specific enrichment of H3K9ac; H3K27me2,3; and H3K9me2, but we also observed a broader epigenetic shift: Histone methylation and acetylation were often decreased and increased, respectively, in EtOH-exposed cells (Fig. 2h, i and Supplementary Table 4). Histone deacetylase and methyltransferase expression were concordantly increased between all cell types in association with EtOH exposure. Moreover, EtOH appeared to induce a systematic decrease in the methylation of promoter regions (Fig. 2h, i). Even though particular histone modifications (namely, those shown in Fig. 2i) showed a statistically significant difference between EtOH-exposed cells and controls, the quantitative difference between particular modifications or patterns of modifications was often subtle, perhaps in reflection of strict epigenetic regulation [43]. Nevertheless, together these findings suggest that EtOH exposure resulted in an epigenetic shift affecting chromatin accessibility and histone post-translational modifications.

### EtOH exposure is associated with transcriptomic alteration in neurodevelopmental pathways

**Fig. 3:**
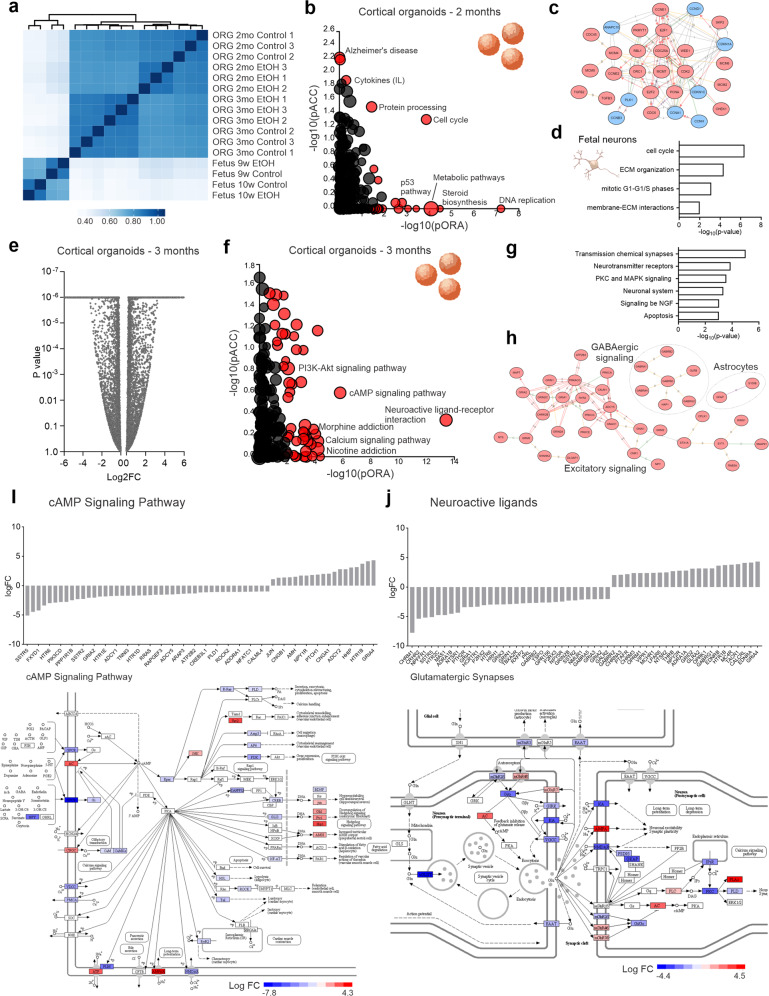
EtOH alters neurodevelopmental transcriptional pathways in cortical organoids and fetal neurons. **a** RNAseq heatmap within cortical organoid and fetal neuron subgroups shows differential gene expression clustering by EtOH exposure. Scale indicates Pearson’s correlation coefficient. Cortical organoids are from the WT83 iPS cell line. **b** Visualization of prominently altered cell processes in two-month old EtOH-exposed organoids. Pathway enrichment analysis of genes altered by EtOH exposure is displayed according to total perturbation accumulation (pACC) and gene overrepresentation within pathways (pORA). Red dots represent pathways modified with a significant *P* value, proportional to the size of the dots. **c** Interactome depicting the interrelatedness of these processes. **d** Cross-comparative gene ontology analysis of EtOH-exposed fetal neurons shows overlapping pathway alterations with organoids. **e** Plot of *P* values for gene expression in three-month old organoids portrays range of alteration severity. **f** Visualization of prominently altered cell processes in three-month old EtOH-exposed organoids. **g** Gene ontology analysis of three-month old organoids shows gene expression alterations prominently affect neurotransmission and biochemical signaling pathways. **h** Interactome highlights involvement of key processes including excitatory and GABAergic signaling and astrocytic expression. **i**, **j** EtOH exposure impacts gene expression in prominent pathways with multifactorial downstream consequences. Shown are EtOH-induced changes in the cAMP signaling pathway (**I**) and glutamatergic synaptic function (**j**).

EtOH-induced alterations in chromatin accessibility are expected to contribute to transcriptomic changes that underlie PAE neural cytopathology. We compared gene expression profiles between control and EtOH-exposed organoids and fetal neurons. Broadly, EtOH-exposed cortical organoids and fetal neurons exhibited differential global gene expression and clustered distinctly from controls (Fig. 3a; Supplementary Fig. 3a, b; and Supplementary Table 5). The affected transcriptomic pathways in cortical organoids include protein processing and cell cycle regulation (Fig. 3b and Supplementary Fig. 3c), also identified by ATAC-seq analysis. Many of the altered processes appeared to interact (Fig. 3c), underscoring the breadth of cellular impairment by EtOH. Summary gene ontology analysis in fetal neurons likewise implicated the effects of EtOH on cell cycle and cytoskeletal structure (Fig. 3d and Supplementary Fig. 3d). EtOH induces alterations in cellular signaling pathways, ligand-receptor interaction, and neurotransmission at a later stage in cortical organoids, including excitatory and GABAergic signaling and astrocytic function (Fig. 3e–h and Supplementary Fig. 3e). In addition, cAMP, PI3K-Akt, and Hedgehog signaling were impaired, leading to alterations in cell viability, calcium homeostasis, synaptic vesicle cycling, and long-term potentiation (Fig. 3i, j and Supplementary Fig. 4).

### Digital spatial profiling portrays differential proteomics of EtOH-exposed cortical organoids

**Fig. 4:**
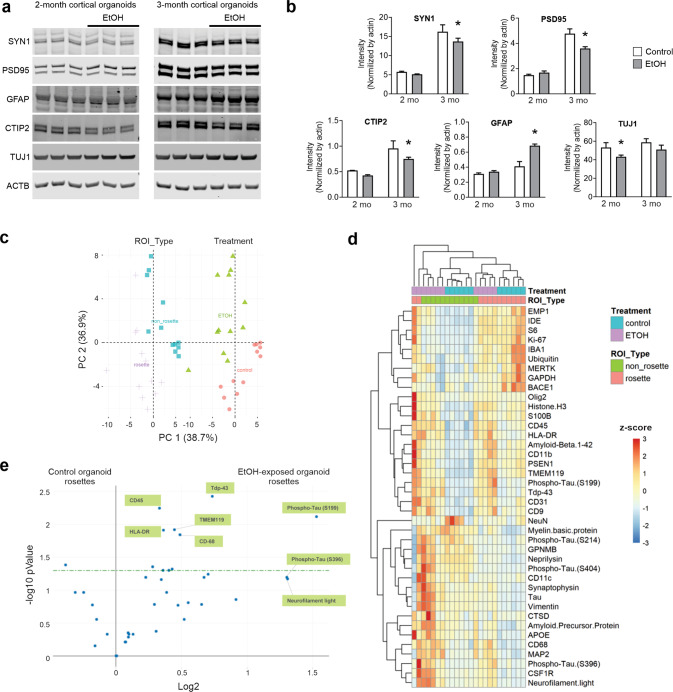
EtOH alters astrocytic and synaptic protein quantities and promotes differential protein abundance in cortical organoids. **a**, **b** Western blot and quantification of astrocytic and synaptic protein markers in cortical organoids during development (two-way ANOVA for each protein; *P < 0.05, **P < 0.01, ***P < 0.001; *n* = 3 replicates (~5–10 organoids)/condition from independent organoid batches from the WT83 iPS cell line). **c** Principal components analysis of protein digital spatial profiling distinguishes distinct clusters between proliferative and non-proliferative regions and between EtOH-exposed and control samples (*n*~10 control and EtOH-exposed orga*n*oids) (**d**) Heatmap showing variation in protein abundance in organoid samples between distinct spatial and EtOH-exposure clusters. **e** Volcano plot of proliferative (rosette) regions from control and EtOH-exposed organoids highlights the differences in expression of various proteins. Green bar represents a *P* value of 0.05.

The combined analysis of epigenetic and transcriptomic profiles implicated synaptic connectivity and astrocytic alteration defects due to EtOH exposure, leading us to target these properties for further investigation. Western blot analysis showed EtOH-exposed cortical organoids exhibited altered content of hallmark neural proteins, including SYN1, PSD95, and GFAP (Fig. 4a, b and Supplementary Fig. 5).

In addition to altered quantity, we sought to further determine whether EtOH exposure affects spatial patterns of neural protein expression within cortical organoids using Digital Spatial Profiling [35], enhancing our analysis with commercial protein panels for neural cell subtyping and cellular neuropathology. Principal components analysis comparing spatial protein expression in cortical organoids distinguished four distinct clusters based on organoid EtOH treatment status (EtOH-exposed vs control) and regional protein localization within the organoid (rosette or non-rosette; Fig. 4c). Interestingly, upon assessing how the expression of particular proteins varies between clusters, it was observed that EtOH-exposed organoids showed a greater abundance of degenerative proteins than controls (Fig. 4d). In particular, proliferative rosette-like regions of EtOH-exposed organoids portrayed enrichment of immune response and degenerative proteins such as Neurofilament light, HLA-DR, CD-45, CD-68, TMEM119, Phospho-Tau (S199) and (S396), and TDP-43 (Fig. 4d, e). In contrast with proliferative rosettes, spatial profiling of other neural proteins, such as synaptophysin, showed the most pronounced differences in the non-rosette cluster (Fig. 4d), corresponding to the more maturely differentiated regions of the organoid. This contrasting spatial expression profile reflects these proteins’ roles in more mature neural cells, such as neurons and glia, leading to further investigation of these cell types.

### EtOH exposure increases astroglia cluster content and impairs synaptogenesis and connectivity

**Fig. 5:**
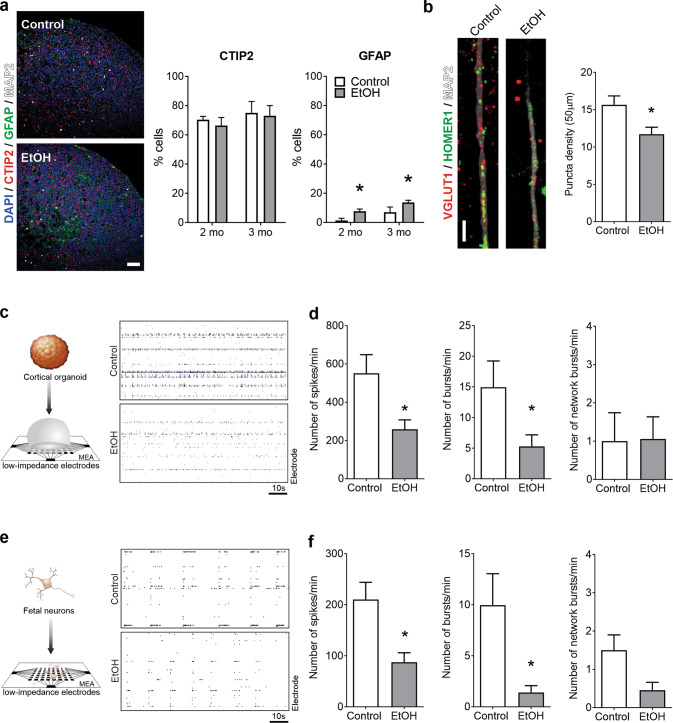
EtOH exposure increases astrocytic content and impairs network connectivity. **a** Immunohistochemistry reveals no difference in the proportion of CTIP2+ cells (left graph; two months, *P* = 0.88; three months, *P* > 0.99; *n* = 3 organoids per age, per condition from WT83 and 4C1 iPS cell lines) but shows EtOH increases GFAP expression (right graph; two months, *P* = 0.017; three months, *P* = 0.013; two-way ANOVAs; *n* = 3 organoids per age, per condition from WT83 and 4C1 iPS cell lines). Scale bar = 50 μm. **b** EtOH exposure decreases co-localized synaptic puncta density (Student’s *t*-test, *t*_32_ = 2.59, *P* = 0.01; *n* = 17 neuro*n*s/condition from WT83, CVB, and 4C1 iPS cell lines). Scale bar = 10 μm. **c**–**e** Multi-electrode array (MEA) analysis in WT83-based cortical organoids (**c**, **d**, *n* = 18 MEA wells/condition) and fetal neurons (**e**, **f**, *n* = 11–22 MEA wells/condition). **c** Representative cortical organoid MEA raster plot. **d** EtOH-exposed cortical organoids showed fewer spikes per minute (Student’s *t*-test, *t*_34_ = 2.68, *P* = 0.011) and fewer bursts (Student’s *t*-test, *P* = 0.047), although not network bursts (Student’s *t*-test, *t*_34_ = 0.06, *P* = 0.95). **e** Representative fetal neuron MEA raster plot. **f** EtOH-exposed fetal neurons showed fewer spikes per minute (Student’s *t*-test, *t*_30_ = 2.68, *P* = 0.012) and fewer bursts per minute (Student’s *t*-test, *t*_40_ = 2.60, *P* = 0.013), but not network bursts (Student’s *t*-test, *t*_31_ = 1.78*, P* = 0.085). Data are presented as mean ± s.e.m.

Given convergence of the epigenetic, transcriptomic, and proteomic findings towards EtOH-induced defects in synaptic connectivity and astrocytic alterations, we sought to gain insights into a potential cellular vulnerability by quantifying cell type in cortical organoids. EtOH exposure was associated with higher percentages of cells in clusters expressing the astrocytic GFAP marker at two (*P* = 0.007) and three months of age (*P* = 0.04; Fig. 5a). Despite the astrocytic increase, the neuronal fate seemed unaffected because quantities of the cortical layer V/VI neuronal marker CTIP2 remained equivalent (Fig. 5a). Impairment of synaptogenesis was evaluated by quantifying synaptic puncta density, characterized by co-localization of the presynaptic Vglut1 and postsynaptic Homer1 proteins. Compared to controls, reduced synaptic puncta density was observed in two-month-old, EtOH-exposed organoids (*P* = 0.01; Fig. 5b), suggesting early EtOH exposure can impact neuronal connectivity for an extended period.

Multi-electrode array electrophysiology recordings enable dynamic interrogation of how human neurons behave in circuits, a valuable approach given that connectivity cannot be assessed during the early stages of human corticogenesis in utero. We plated one-month-old paired control and EtOH-exposed organoids to mature in MEA plates and recorded their activity (Fig. 5c, e). Compared to controls, EtOH-exposed cortical organoids spiked fewer times per minute (*P* = 0.01) and had fewer bursts (*P* = 0.047), although synchronous network bursts remained unchanged (*P* = 0.93; Fig. 5d). Finally, we used primary human fetal neural tissues to validate our results. EtOH-exposed fetal neurons exhibited a similar MEA profile with fewer spikes (*P* = 0.01) and fewer bursts (*P* = 0.01) per minute, but the decrease in synchronous network bursts did not reach significance (*P* = 0.08; Fig. 5f). These findings showed that EtOH-induced alterations in neuronal network formation persist despite cessation of the exposure.

### Pharmacological reversal of synaptic deficits associated with EtOH exposure

**Fig. 6:**
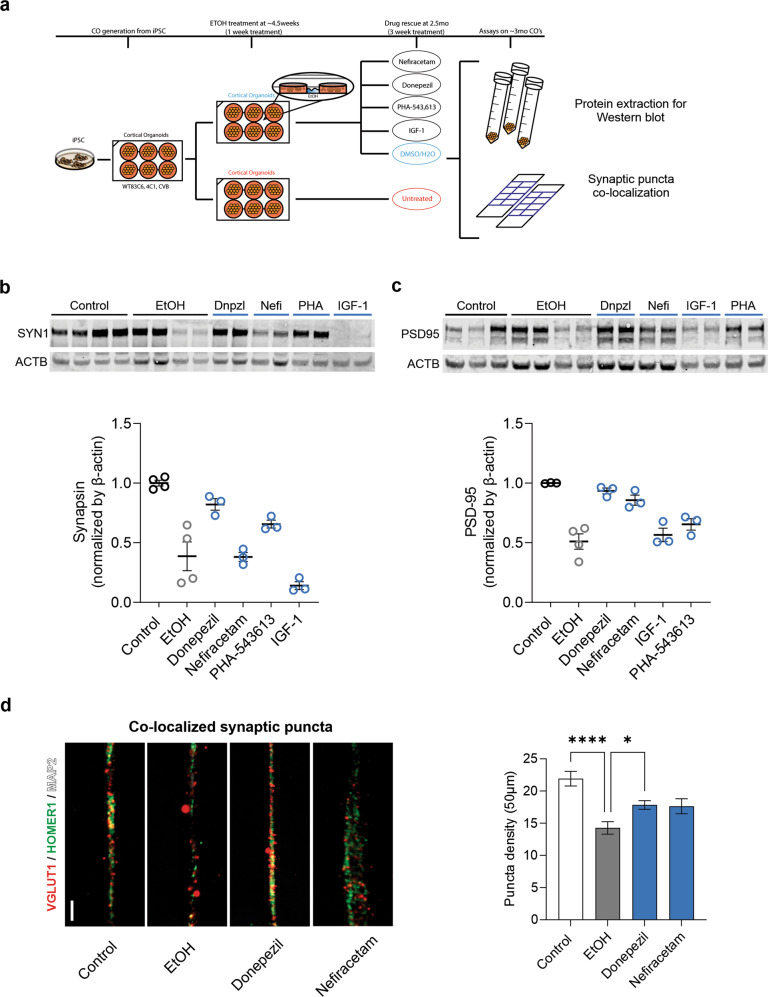
Pharmacological reversal of synaptic impairment associated with EtOH exposure. **a** Schematic showing the drug treatment strategy of EtOH-exposed cortical organoids. **b** Western blot shows a reduced amount of the presynaptic protein Synapsin in EtOH-exposed organoids that is increased by treatment with Donepezil, but not Nefiracetam (Nefi) or IGF-1. **c** Levels of the postsynaptic protein PSD-95 were rescued by treatment with either Donepezil or Nefiracetam, but not IGF-1. **d** Donepezil treatment reversed the synaptic puncta impairment observed in EtOH-exposed organoids (one-way ANOVA with Dunnett’s test for multiple comparisons, *F*_3,173_ = 10.86, *P* < 0.0001; Control vs EtOH, *P* < 0.0001; Donepezil vs EtOH, *P* = 0.024; *n* = 48 neurites/conditio*n* from WT83, CVB, and 4C1 iPS cell lines), with lesser support for the efficacy of Nefiracetam (*P* = 0.066; *n* = 33 neurites from WT83 and CVB iPS cell lines). Dnpzl Donepezil. Nefi Nefiracetam. Data are presented as mean ± s.e.m.

Having identified a series of pathophysiological changes in diverse human neural cell models exposed to EtOH, we sought to investigate whether pharmacological intervention could ameliorate the severity of the synaptic phenotypes (given their prominent representation in our findings) utilizing an abridged version of the drug screening pipeline strategy recently shown by our group to be effective [26]. Four drugs were selected for screening in EtOH-exposed organoids (Fig. 6a) based on their predicted ability to rescue synaptic impairment: Nefiracetam, PHA-543613, Donepezil, and IGF-1. Nefiracetam and PHA-543613 were chosen because our group has previously demonstrated their ability to reverse synaptic impairment associated with disease [26]. Furthermore, a compound from the same racetam class of drugs in which Nefiracetam is included, aniracetam, has previously been shown to reverse neurocognitive deficits in a mouse model of PAE by modulating synaptic transmission [44–46], bolstering support for trial of Nefiracetam in our human cell models of PAE. Donepezil, a compound clinically indicated as first-line treatment to slow cognitive decline and dementia, was selected because chronic alcohol usage is associated with dementia and Alzheimer’s disease, and we identified changes in neurodegenerative proteins (e.g., phospho-tau isoforms and Aβ_1-42_) in proteomic spatial profiling in EtOH-exposed compared to control organoids.

Western blot of synaptic proteins and synaptic puncta co-localization were used as pilot assays to evaluate the drug candidates’ potential to reverse synaptic phenotypes observed in EtOH-exposed organoids (Fig. 6a). Each of the four drug candidates was assessed via Western blot of synaptic proteins. Only Donepezil appreciably increased the amount of presynaptic SYN1 in EtOH-exposed organoids (Fig. 6b), although PSD-95 was notably increased by treatment with either Donepezil or Nefiracetam (Fig. 6c). Donepezil and Nefiracetam were subsequently evaluated for their potential to reverse synaptic puncta impairment in EtOH-exposed organoids (*P* < 0.0001; Fig. 6d). Co-localized puncta density in EtOH-exposed organoids was increased by treatment with Donepezil (*P* = 0.024; Fig. 6d). Although the increase from treatment with Nefiracetam did not reach statistical significance (*P* = 0.066; Fig. 6d), the 95% confidence interval (CI) for its effect nearly completely overlaps that of Donepezil (95% CI: EtOH vs. Donepezil, [−6.75, −0.37]; EtOH vs. Nefiracetam, [−6.89, 0.17]).

## Discussion

In the present study, hiPSC-derived cortical organoids were exposed to EtOH and then comprehensively assayed to investigate the neurodevelopmental impact of EtOH on neural proliferation and viability, chromatin alteration and histone modification, transcriptomic and proteomic shifts, and neuronal electrophysiology and network formation. We validated our findings and further explored cell-type specific defects in hiPSC-derived astrocytes and human fetal primary neurons. EtOH-exposed cortical organoids showed impaired proliferation and increased cell death, and we observed shifts in their epigenomic and transcriptomic profiles resulting from the exposure that paralleled the particular cell-type-specific defects that we had detected. In particular, the regions of altered chromatin accessibility were most prominently related to neurodevelopment, neurotransmission, cell-cell interaction, and Rho GTPase signaling. Concordantly, electrophysiological analysis demonstrated that EtOH exposure caused prolonged functional impairment and decreased network connectivity in the organoids that was validated by findings in human fetal primary neurons.

Prenatal exposure to EtOH is associated with microcephaly during all stages of brain growth [47–49], and magnetic resonance imaging (MRI) in FASD patients portrays volumetric reductions in the cortex, corpus callosum, cerebellum, and other subcortical structures [50–53]. Similarly, we observed that the size of EtOH-exposed cortical organoids was reduced compared to control organoids. In agreement, EtOH-exposed organoids had fewer dividing cells during proliferative stages and increased cell death, a phenotype that aligns with effects reported in other model systems [54, 55]. However, despite this convergence, it is unlikely that these acute neurotoxic effects are the only pathologies underlying the long-lasting effects of PAE.

Epigenetic signaling mediates the transcriptional response to environmental stimuli, and EtOH has previously been shown to induce epigenetic alterations [56]. ATAC-seq of organoids showed that EtOH exposure was associated with widespread changes in chromatin accessibility that was most prominently related to neurodevelopment, neurotransmission, and Rho GTPase signaling. Rho GTPases have been linked with gene expression, cell–cell interaction, neuronal morphology, and cell cycle progression [57, 58], and they also contribute to the regulation of neuronal survival via MAPK pathway activation. Additionally, a decreased accessibility of chromatin sites related to cell interaction was observed, such as the neurexin genes. The neurexins are involved in early cortical synaptogenesis, axon guidance, and intercellular communication, and mutations in *NRXN3* have been identified in patients with autism spectrum disorders [59–62]. Furthermore, we used the GeoMx DSP platform to investigate the spacial profile of proteins in specific regions of the organoids. Interestingly, EtOH exposure affected numerous proteins that are salient in other neurological diseases, including inflammatory and degenerative proteins (Neurofilament light, Phospho-Tau (S396), HLA-DR, CD-45, CD-68, TMEM119, and TDP-43). The cell models used in this study lack neuroinflammatory cells and are thus sub-optimal to more deeply study the cell-type specific aspects of these pathologies, so further investigation of these findings using other models will be an important direction for future research.

DNA methylation and histone modifications regulate the accessibility of DNA for transcriptional proteins and are thought to be central mediators of the lifelong clinical effects of FASD [63]. Using mass spectrometry, we quantified the relative abundance of >80 different post-translational histone modifications (e.g., acetylation, methylation, ubiquitination, and unmodified peptides). EtOH exposure induced a unique profile of histone modifications among cortical organoids, hiPSC-derived astrocytes, and human fetal neurons. Notably, EtOH exposure produced parallel duration-dependent gradients in the modification patterns in cortical organoids and human fetal neurons (e.g., acetylation and H3K14UN, H3K79ME1 and H4K12AC and H3K23). These findings align with previously reported EtOH-induced changes in post-translational modifications in FASD patients and other model systems [64, 65], further underscoring their involvement in its pathophysiology.

Aligning epigenetic information with transcriptomic data may indicate how EtOH affects early brain formation. Exposed samples showed differences in gene expression and clustered distinctly from controls. In early cortical organoids, the main processes affected by EtOH exposure were related to normal cellular function, such as protein processing and cell cycle. However, later in their development, transcriptional alterations were related to cAMP signaling, neuroactive ligand pathways, RAP1, and Hippo signaling pathways. The Hippo signaling pathway regulates growth via proliferation and apoptosis, which may partially explain the EtOH-induced cellular phenotypes we observed.

Beyond its neuronal effects, PAE may also impair neurodevelopment via astrocytic disruption. EtOH increased cell death, altered the histone modification pattern, and produced shifts in the astrocytic cell cycle and proliferation in parallel with the findings of other studies [66, 67]. Interestingly, the amount of astrocytic protein GFAP was increased in EtOH-exposed organoids, suggesting that astrocytes may be more involved with PAE pathogenesis than was previously appreciated. Indeed, consistent with our findings, a previous study by Boronat and coworkers [68] detected abundant gliosis in MRIs of 72 pediatric FASD patients. Further investigation of astrocytic involvement in the setting of PAE will be an important direction for future studies.

We performed MEA electrophysiological analysis of neuronal network circuitry and validated our findings using human primary neurons. EtOH exposure impaired synaptogenesis, concordant with gross and microscopic neuropathology described in FASD patients [69]. These findings contrast with the propensity for seizures in these patients and murine models, which is thought to result from neuronal hyperexcitability. However, comparing these differences may be inequitable because these observations are drawn from hippocampal regions, whereas our models are cortical [70, 71]. Nevertheless, the induced network changes persisted for months despite halting the exposure, supporting the prevailing understanding that even acute EtOH exposure is sufficient to produce lasting neurodevelopmental effects.

Although this study is a step towards understanding PAE, it was unavoidably subject to several limitations. Despite advancements in technology, cortical organoids are only a cellular model rather than a bonafide human brain, so making a direct judgment about the full effects of *in utero* EtOH exposure remains limited. We have sought to minimize this limitation by including analyses in human primary fetal neurons, which appear to validate the findings in the cortical organoids. Another limitation is that EtOH metabolites were not assessed. In consequence, although EtOH exposure is associated with specific cellular alterations, it is unclear whether particular metabolites or variations in the rate of EtOH metabolism, for example, can alter the severity of the effects of the exposure. However, our results were consistent between cell lines and exposures, minimizing any concern about individual metabolite variation. A third limitation is that the EtOH concentration and exposure are unavoidably different from those experienced during fetal development, particularly considering fetal recycling of amniotic fluid and the consequent risk of prolonged EtOH exposure in utero [72].

Although many of the cellular changes we observed in association with EtOH exposure agree with findings documented in human clinical studies of PAE or animal models, the present study is unable to determine whether the changes we detected are specific to EtOH or if they could be induced by other toxins. Similarly, the multifactorial pathophysiology of EtOH exposure, which depends not merely on duration and dosage of exposure but also on complex interactions between genetic and environmental variables and their brain cell-type specific effects, precludes precise definition of a causally specific mechanism linking EtOH exposure with particular cytological abnormalities. Precisely refining the EtOH-specific pathophysiology in human cell models will be an important objective for future research. As an additional limitation, although many of the experiments in the present study utilized both male and female iPS cell lines, we are unable to ascribe specific effects to sex due to the low sample size, hindering our ability to identify any sex-specific differences of EtOH exposure. Nevertheless, the possibility of such effects warrants further investigation in future studies.

The pharmacological rescue experiments are further subject to another important limitation. Although we rigorously selected compounds based on our experience [26], our findings should be regarded as preliminary evidence that, prior to decisive interpretation, require validation in future studies.

## Conclusion

hiPSC-derived cortical organoids are a valuable model to assess the effects of EtOH exposure on human neurodevelopment and will facilitate further study of the pathophysiology underlying the clinical symptoms associated with PAE. Supported by validation in human primary fetal neurons, EtOH-exposed cortical organoids portrayed impaired cell growth and viability, featured alterations in their epigenomic and transcriptomic profiles in regions critical to neurodevelopment, and experienced dysfunctional neuronal network formation. EtOH exposure disrupted several prominent growth and signaling pathways, and future research efforts may discover that these are opportune targets to therapeutically impede the developmental neuropathology of PAE.

## Supplementary information

Supplementary Material

Supplementary Figure 1

Supplementary Figure 2

Supplementary Figure 3

Supplementary Figure 4

Supplementary Figure 5

Supplementary Table S1_neurodevelopmental marker genes

Supplementary Table S2_ATACseq controls_only

Supplementary Table S3_ATACseq EtOH_only

Supplementary Table S4_mod spec

Supplementary Table S5_RNAseq
