# Supplementary material for: Impact of alcohol exposure on neural development and network formation in human cortical organoids
Source: Mol Psychiatry. 2022 Nov 16;28(4):1571–84. doi: 10.1038/s41380-022-01862-7 (PMC10208963; doi:10.1038/s41380-022-01862-7)
Supplement: Supplementary file 1 — Supplementary Material [file 41380_2022_1862_MOESM1_ESM.doc]

**Supplementary Table Legends**

**Supplementary Table 1. Regional marker genes of neurodevelopment.**

**Supplementary Table 2. ATAC sequencing showing peaks present in control cortical organoids.**

**Supplementary Table 3. ATAC sequencing showing peaks present in EtOH-exposed cortical organoids.**

**Supplementary Table 4. Relative abundance of histone post-translational modification of a given tryptic peptide, and standard deviation of corresponding measurements from three mass spec runs.** UN – Unomodified; ME1/2/3 - Mono/di/tri methylation; AC – Acetylation and UB – Ubiquitination.

**Supplementary Table 5. Global RNA sequencing expression data of cortical organoids exposed to EtOH.**

**Supplementary Figure Legends**

**Supplementary Figure 1. Generation and characterization of iPSC-derived cortical organoids and astrocytes and characterization of fetal human primary neurons. a** EtOH concentration in media sharply decreases in the first two hours and stabilizes around 20 mM with time. EtOH was added every other day. **b** Immunohistochemical characterization of neural markers in young cortical organoids. **c** Cell type as a percentage changes with the progression of cortical organoid development. **d** Heatmap visualization of hierarchical clustering of organoid sample gene markers correlated with BrainSpan reference human brain transcriptome reveals a transcriptomic profile of organoids that closely aligns with gene markers of cortical identity. **e** Quantification of astrocytic population purity. **f,g** Representative plots for annexin (**f**) and cell cycle (**g**) between control and EtOH-exposed astrocytes. **h** Quantification of fetal primary neuron population purity. i,**j** Representative plots for annexin (**i**) and cell cycle (**j**) between control and EtOH-exposed fetal neurons. CTC, cortical organoid; OFC, orbital prefrontal cortex; DFC, dorsolateral prefrontal cortex; VFC, ventrolateral prefrontal cortex; MFC, medial prefrontal cortex; M1C, primary motor (M1) cortex; S1C, primary somatosensory (S1) cortex; AMY, amygdaloid; STR, striatum; ganglio, ganglionic eminence; MGE, medial ganglionic eminence; LGE, lateral ganglionic eminence; CGE, caudal ganglionic eminence; DTH, dorsal thalamus; STC, superior temporal cortex; ITC, inferior temporal cortex; HIP, hippocampus; PCx, parietal cortex; TCX, temporal cortex; OCX, occipital cortex; CB, cerebellum.

**Supplementary Figure 2. ATAC-seq peak distribution and mass spectrometry analysis of histone modification in cortical organoids due to one day of EtOH exposure. a,b** ATAC-seq plots show the fraction of reads within peaks of enrichment (**a**) and how peaks distribute by genomic region (**b**). **c** Autocorrelation plots for samples. **d,e** Plots showing peak regions (**d**) and TS sites concentrated around promoters (**e**).

**Supplementary Figure 3. RNA-seq analysis of control and EtOH-exposed cortical organoids and fetal neurons. a** Samples segregate with distinct gene expression signatures. **b** Visualization of the genomic distribution of reads. **c** Gene expression comparison between EtOH-exposed and control two-month-old cortical organoids. **d** Gene expression comparison between EtOH-exposed and control fetal neurons. CTC, cortical organoid; HU, human fetal primary neurons.

**Supplementary Figure 4. Pathway analyses of EtOH-induced differential gene expression in cortical organoids. a** Broad involvement of pathways relevant to neural development and function. **b** Prominent involvement of calcium signaling pathways, with central consequences in MAPK signaling, long-term potentiation and depression, apoptosis, and the phosphatidylinositol signaling pathway. **c** EtOH-induced changes in gene expression include effects on the TGF-β and Wnt signaling pathways as well as cell-cell interactions via adherens junctions. **d** EtOH-induced changes in the RAP1 signaling pathway are likewise consequential for calcium signaling, cell-cell adhesion, and MAPK signaling.

**Supplementary Figure 5. Protein level analysis in different developmental time points and regions in cortical organoids. a** Full Western blots shown for two-month and three-month-old control and EtOH-exposed cortical organoids. **b** A normalizing signal-to-noise ratio (SNR) plot for for digital spatial profiling isolates the most relevant proteins as those with values above the SNR > 3 threshold. Protein values below SNR < 1 may be attributed to background noise, and proteins with SNR values between 1 and 3 may need further validation. **c** Digital spatial proteomic profile of Ki67 and MAP2 staining of selected proliferative (rosette) and differentiated (non-rosette) regions within each cortical organoid condition. **d** Plot showing Ki67 expression for all selected areas of interest (AOIs) corresponding to stain images in (**b**)**.**

**Supplementary Materials and Methods**

*Generation of cortical organoids*

Cortical organoids were generated as previously described^1–3^. The newly formed cortical organoids were maintained in defined culture media transitioning through factors to promote proliferation, maturation, gliogenesis, and electrophysiological activity. Briefly, hiPSC colonies were dissociated with Accutase (Life Technologies) and resuspended in mTeSR1 supplemented with 10 μM SB431542 (SB; Stemgent, Cambridge, MA, USA), 1 μM Dorsomorphin (Dorso; R&D Systems, Minneapolis, MN, USA), and 5 μM ROCK inhibitor (Y-27632; Calbiochem, Sigma-Aldrich, St. Louis, MO, USA). After three days, media was switched to Media1 [Neurobasal (Life Technologies) supplemented with 1X GlutaMAX (Life Technologies), 1X Gem21 (Gemini Bio-Products), 1X N2 NeuroPlex (Gemini Bio-Products), 1X NEAA (Life Technologies), 1X Pen/Strep (Life Technologies), 10 μM SB, and 1 μM Dorso] for six days. Media2 [Neurobasal with 1X GlutaMAX, 1X Gem21, 1X NEAA, and 1X Pen/Strep] supplemented with 20 ng/mL FGF2 (Life Technologies) was used for seven days and then further supplemented with 20 ng/mL EGF (PeproTech, Rocky Hill, NJ, USA) with 20 ng/mL FGF2 every other day for another six days. Next, cells were maintained for six days in Media3 [Media2 supplemented with 10 ng/mL BDNF, 10 ng/mL GDNF, 10 ng/mL NT-3 (all from PeproTech), 200 μM L-ascorbic acid, and 1 mM dibutyryl-cAMP (Sigma-Aldrich)]. Cortical organoids were subsequently kept in Media2 alone with media changes every 2-3 days.

*Generation of astrocytes*

Astrocytes were generated according to protocols published elsewhere^4^. Briefly, neural progenitor cells were dissociated with Accutase, transferred to a 6-well plate, and kept in suspension under rotation in neural media [DMEM/F12 (Life Technologies) supplemented with 1X GlutaMAX, 1X Gem21 (Gemini Bio-products), and 1X Pen/Strep (Life Technologies)] containing 20 ng/mL FGF2 (PeproTech). Approximately 5 x 10^6^ cells were plated into each well. ROCK inhibitor (Y-27632; Calbiochem) was added to a final concentration of 5 μM for 48 hours. After the removal of ROCK inhibitor, neural media without FGF2 was used for one week. Next, the media was switched to astrocyte growth medium (AGM; Lonza Group, Basel, Switzerland) for two weeks, when the spheres were plated onto poly-L-ornithine/laminin pre-coated dishes. The spheres continued to be cultured in AGM as astrocytes projected outward to populate the plate.

*Gene expression profile (RNA sequencing)*

Total polyA-based RNA was extracted from treated and non-treated cells using the RNeasy Kit (Qiagen, Hilden, Germany). Extracted RNA was prepared as stranded RNAseq libraries using Truseq Stranded mRNA LT Kit (Illumina, San Diego, CA, USA) and was multiplexed and sequenced on Illumina HiSeq 2500 to generate 100 bases paired-end reads, considering a minimum of 40 million of sequenced fragments per sample. Data were analyzed by Rosalind (https://rosalind.onramp.bio/), with a HyperScale architecture developed by OnRamp BioInformatics, Inc. (San Diego, CA, USA). Reads were trimmed using cutadapt, and quality scores were assessed using FastQC. Reads were aligned to the *Homo sapiens* genome build hg19 using STAR. Individual sample reads were quantified using HTseq and normalized via Relative Log Expression (RLE) using DESeq2 R library. Functional enrichment analysis of pathways, gene ontology, domain structure, and other ontologies were performed using HOMER^5^. Several database sources were referenced for enrichment analysis, including Interpro^6^, NCBI, MSigDB^7,8^, REACTOME^9^, WikiPathways^10^, and Advaita^11^. Enrichment was calculated relative to a set of background genes relevant to the experiment.

*Cortical organoid transcriptomic comparison to developing human brain*

The transcriptomic expression profile from the BrainSpan spatiotemporal atlas of human brain development^12^ was retrieved (https://www.brainspan.org/). Using our RNA sequencing data in control and EtOH-exposed organoids, raw count RNA transcript levels were quantified as Transcripts Per Kilobase Million (TPM) using Salmon^13^. Spearman correlation was calculated for each sample transcriptome (all genes with FPKM/RPKM/TPM > 1) compared with bulk transcriptome data from the BrainSpan reference datasets; expected developing brain regions and stages are presented with a correlation matrix using the corrr package for R studio^14^. The data was subset by commonly used gene markers (Supplementary Table 1) for developing brain drawn from the literature^15–17^, Spearman correlations were recalculated. Organoid sample marker genes underwent hierarchical clustering using Euclidean distance, and samples were clustered by Pearson’s correlation with the reference datasets using the log-transformed counts Z-score (mean-centering and dividing by standard deviation). Heatmap visualization was produced with ComplexHeatmap for R^14^.

*Immunofluorescence staining*

Cells were fixed with 4% paraformaldehyde, permeabilized and blocked with 0.1% Triton X-100 and 3% FBS in PBS, and incubated with primary antibodies overnight at 4ºC. Primary antibodies used in this study were: rat anti-CTIP2, ab18465 (Abcam, Cambridge, United Kingdom), 1:500; rabbit anti-cleaved-caspase-3, #9661 (Cell Signaling, Danvers, MA, USA), 1:400; chicken anti-MAP2, ab5392 (Abcam), 1:2000; mouse anti-NeuN, MAB377 (EMD-Millipore, Burlington, MA, USA), 1:500; rabbit anti-Ki67, ab15580 (Abcam), 1:1000; rabbit anti-GFAP, Z033429 (DAKO A/S, Glostrup, Denmark), 1:1000; mouse anti-Vglut1, 135311 (Synaptic Systems, Goettingen, Germany), 1:500; rabbit anti-Homer1, 160003 (Synaptic Systems), 1:500. After being washed with PBS, samples were incubated with secondary antibodies (Alexa Fluor 488-, 555- and 647-conjugated antibodies, Life Technologies, 1:1000) for two hours at room temperature. The slides were mounted using ProLong Gold antifade reagent (Thermo Fisher Scientific, Waltham, MA, USA) and analyzed under a fluorescence microscope.

*Western blotting*

Western blotting was performed as previously described^18^. Total protein was extracted from cortical organoids and quantified with the BCA Protein Assay Kit (Pierce Biotechnology, Rockford, IL, USA). After separation, membrane transfer, and blocking of 20μg of protein, membranes were incubated with primary antibodies overnight at 4ºC [rabbit anti-Synapsin1, AB1543P (EMD-Millipore), 1:500; rabbit anti-GFAP, Z033429 (DAKO A/S), 1:1000; mouse anti-PSD95 NeuroMab, 1:1000; rabbit anti-cleaved-caspase-3, #9661 (Cell Signaling), 1:400; mouse anti-β-III-tubulin, MMS-435P (Covance, Princeton, NJ, USA), 1:1000; rat anti-CTIP2, ab18465 (Abcam), 1:500; mouse anti-β-Actin,  ab8226 (Abcam), 1:7000], followed by two-hour incubation with secondary antibodies before imaging and quantification using the Odyssey CLx imaging system (Li-Cor, Lincoln, NE, USA).

**References**

1. Trujillo, C. A. *et al.* Complex Oscillatory Waves Emerging from Cortical Organoids Model Early Human Brain Network Development. *Cell Stem Cell* **25**, 558-569.e7 (2019).

2. Negraes, P. D. *et al.* Altered network and rescue of human neurons derived from individuals with early-onset genetic epilepsy. *Mol. Psychiatry* **26**, 7047–7068 (2021).

3. Trujillo, C. A. *et al.* Reintroduction of the archaic variant of NOVA1 in cortical organoids alters neurodevelopment. *Science (80-. ).* **371**, (2021).

4. Santos, R. *et al.* Differentiation of Inflammation-Responsive Astrocytes from Glial Progenitors Generated from Human Induced Pluripotent Stem Cells. *Stem Cell Reports* **8**, 1757–1769 (2017).

5. Heinz, S. *et al.* Simple Combinations of Lineage-Determining Transcription Factors Prime cis-Regulatory Elements Required for Macrophage and B Cell Identities. *Mol. Cell* **38**, 576–589 (2010).

6. Mitchell, A. L. *et al.* InterPro in 2019: Improving coverage, classification and access to protein sequence annotations. *Nucleic Acids Res.* **47**, D351–D360 (2019).

7. Liberzon, A. *et al.* Molecular signatures database (MSigDB) 3.0. *Bioinformatics* **27**, 1739–1740 (2011).

8. Subramanian, A. *et al.* Gene set enrichment analysis: A knowledge-based approach for interpreting genome-wide expression profiles. *Proc. Natl. Acad. Sci. U. S. A.* **102**, 15545–15550 (2005).

9. Fabregat, A. *et al.* The Reactome Pathway Knowledgebase. *Nucleic Acids Res.* **46**, D649–D655 (2018).

10. Slenter, D. N. *et al.* WikiPathways: A multifaceted pathway database bridging metabolomics to other omics research. *Nucleic Acids Res.* **46**, D661–D667 (2018).

11. Draghici, S. *et al.* A systems biology approach for pathway level analysis. *Genome Res.* **17**, 1537–1545 (2007).

12. Kang, H. J. *et al.* Spatio-temporal transcriptome of the human brain. *Nature* **478**, 483–489 (2011).

13. Patro, R., Duggal, G., Love, M. I., Irizarry, R. A. & Kingsford, C. Salmon provides fast and bias-aware quantification of transcript expression. *Nat. Methods* **14**, 417–419 (2017).

14. Team, R. C. R: A language and environment for statistical computing. (2020).

15. Fietz, S. A. *et al.* Transcriptomes of germinal zones of human and mouse fetal neocortex suggest a role of extracellular matrix in progenitor self-renewal. *Proc. Natl. Acad. Sci. U. S. A.* **109**, 11836–11841 (2012).

16. Stein, J. L. *et al.* A quantitative framework to evaluate modeling of cortical development by neural stem cells. *Neuron* **83**, 69–86 (2014).

17. Camp, J. G. *et al.* Human cerebral organoids recapitulate gene expression programs of fetal neocortex development. *Proc. Natl. Acad. Sci.* **112**, 201520760 (2015).

18. Thomas, C. A. *et al.* Modeling of TREX1-Dependent Autoimmune Disease using Human Stem Cells Highlights L1 Accumulation as a Source of Neuroinflammation. *Cell Stem Cell* **21**, 319-331.e8 (2017).
