# Supplementary figures and images for: Impact of alcohol exposure on neural development and network formation in human cortical organoids
Source: Mol Psychiatry. 2022 Nov 16;28(4):1571–84. doi: 10.1038/s41380-022-01862-7 (PMC10208963; doi:10.1038/s41380-022-01862-7)

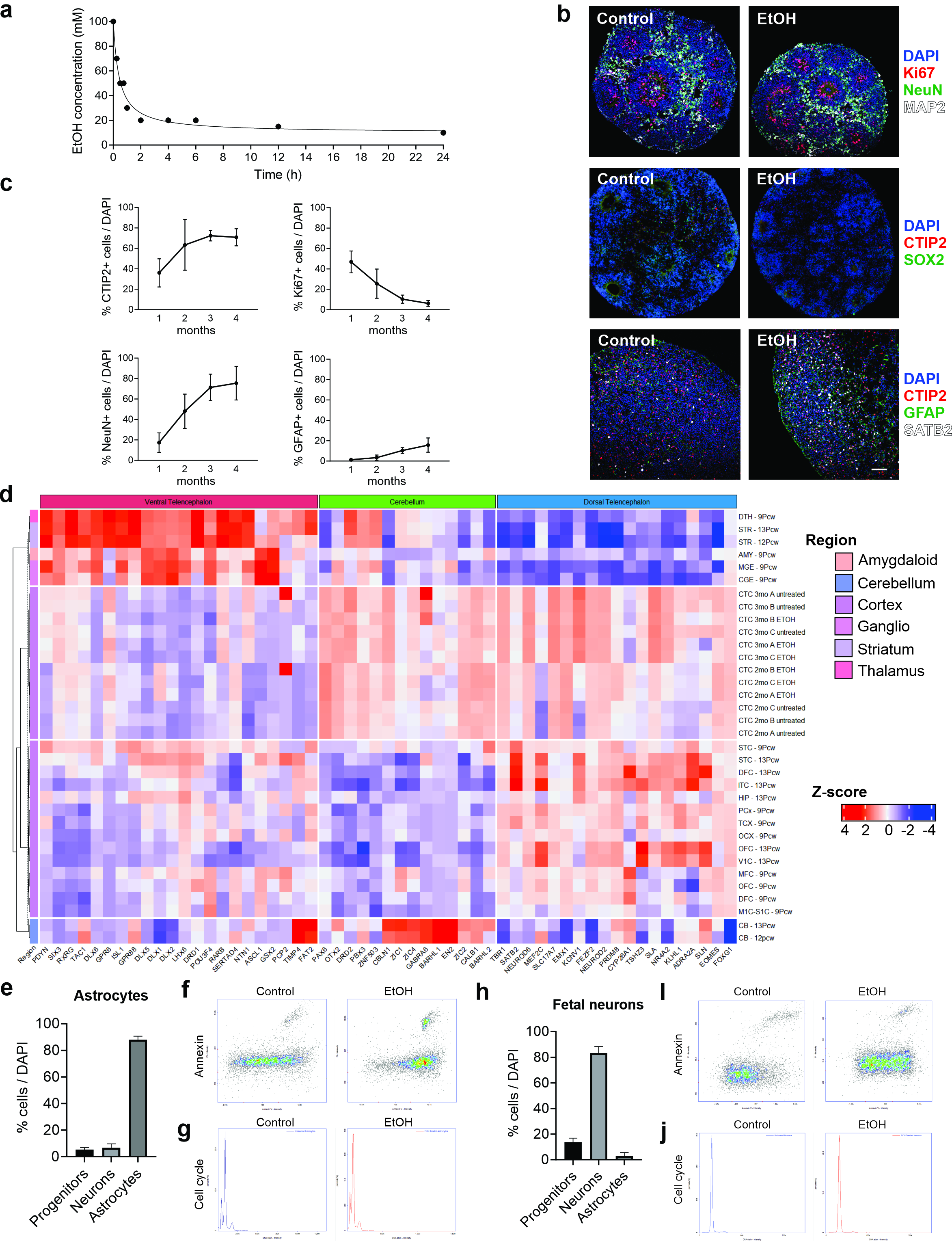

Supplement: Supplementary file 2 — Supplementary Figure 1 [file 41380_2022_1862_MOESM2_ESM.tif]

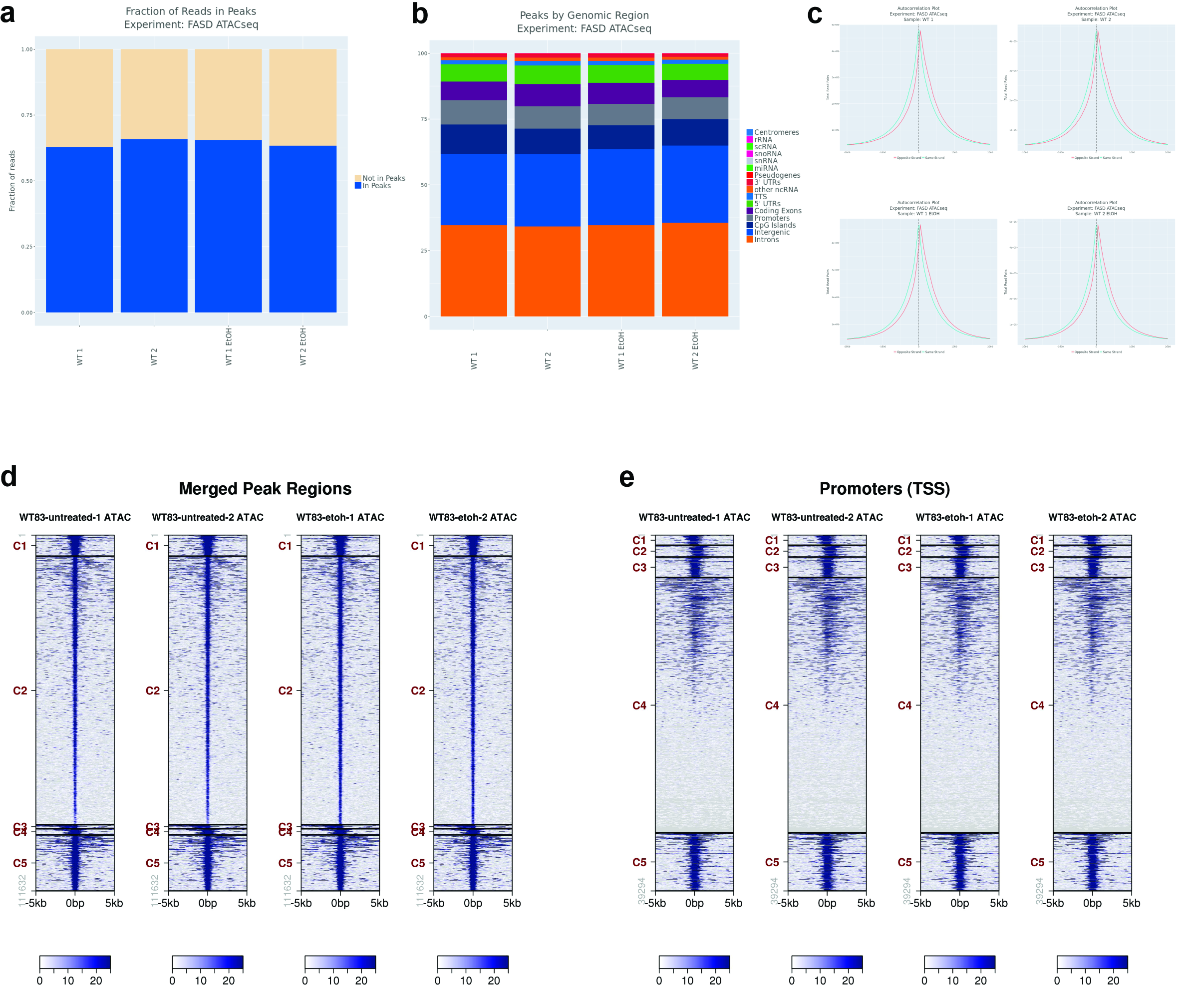

Supplement: Supplementary file 3 — Supplementary Figure 2 [file 41380_2022_1862_MOESM3_ESM.tif]

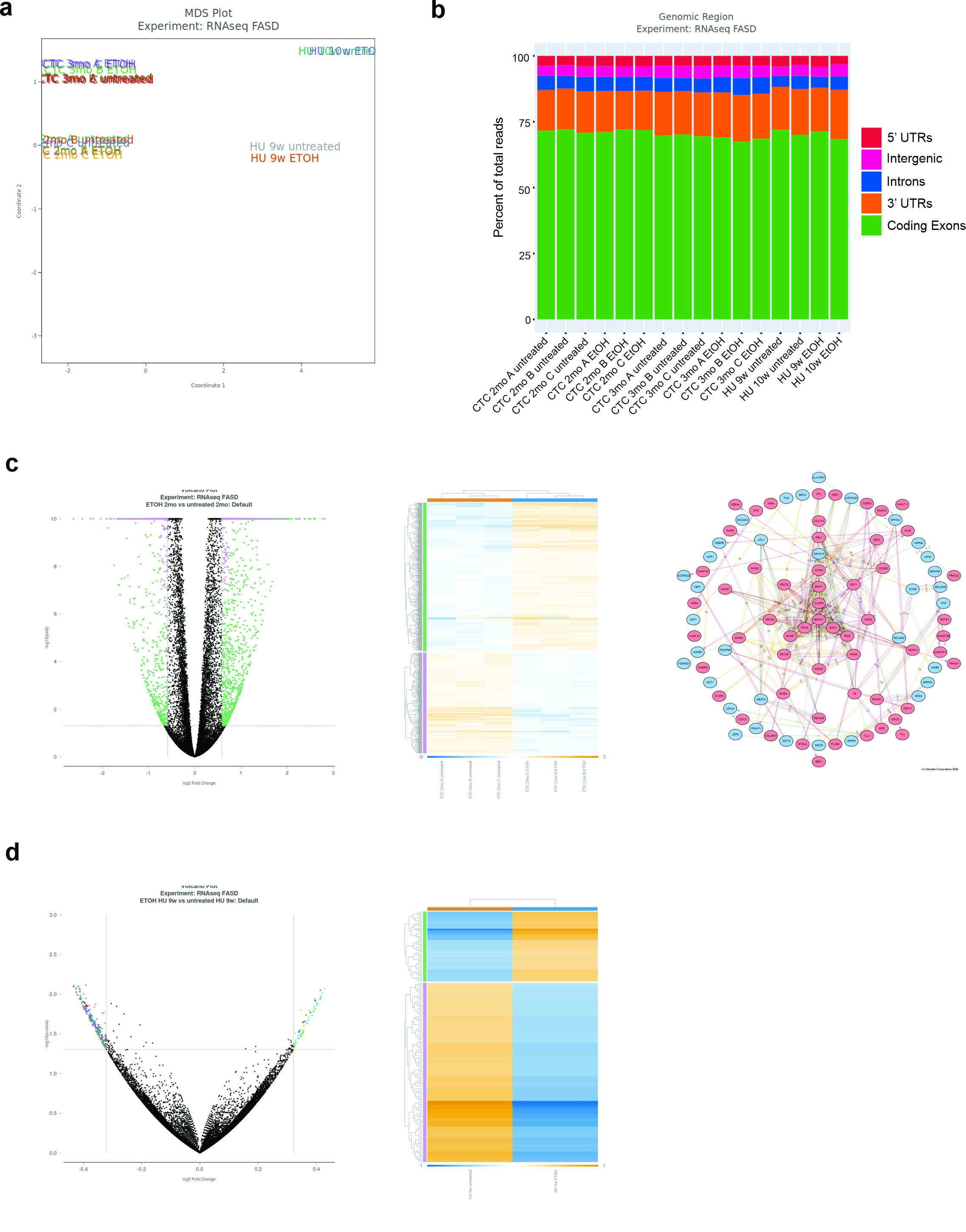

Supplement: Supplementary file 4 — Supplementary Figure 3 [file 41380_2022_1862_MOESM4_ESM.tif]

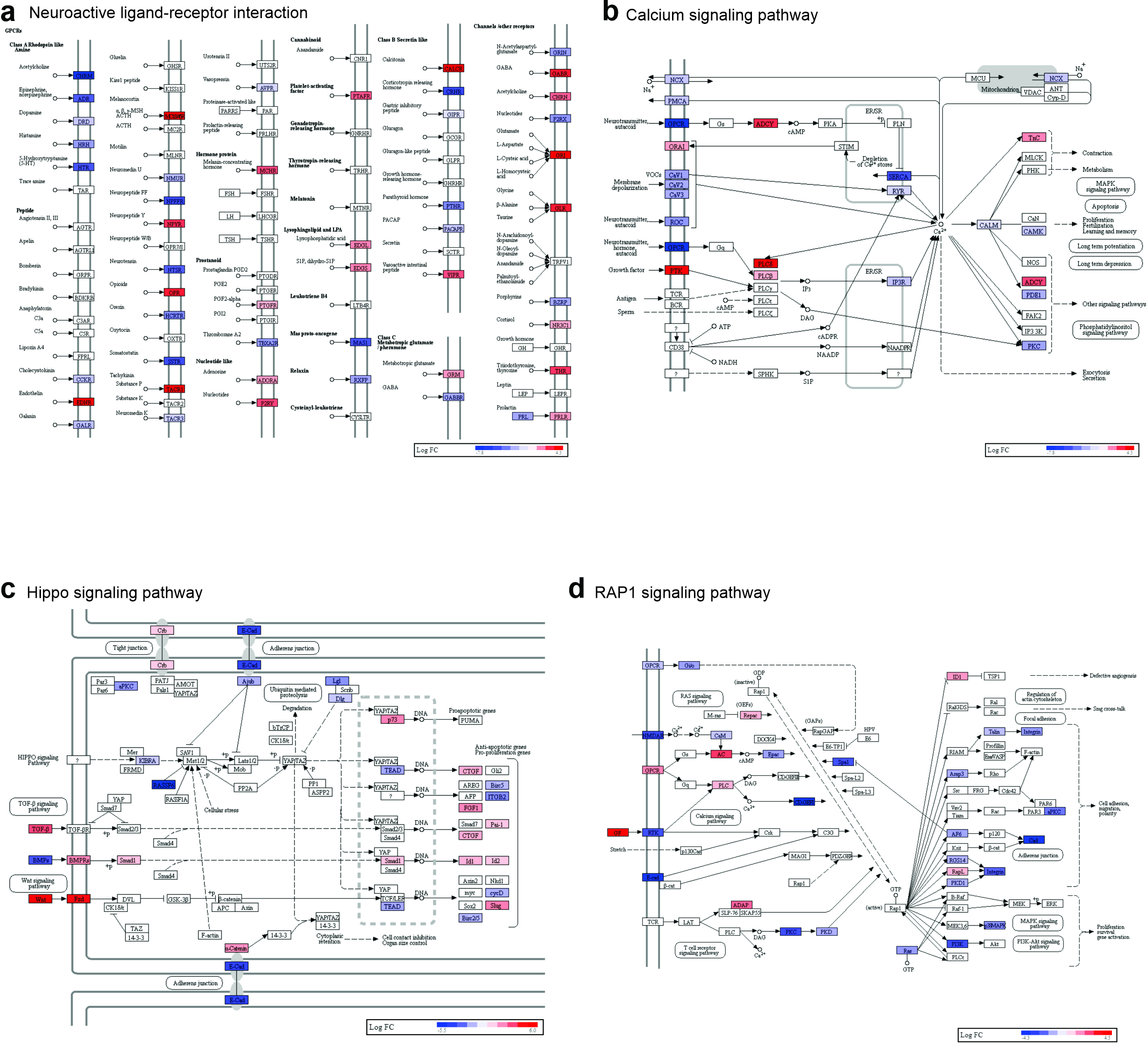

Supplement: Supplementary file 5 — Supplementary Figure 4 [file 41380_2022_1862_MOESM5_ESM.tif]

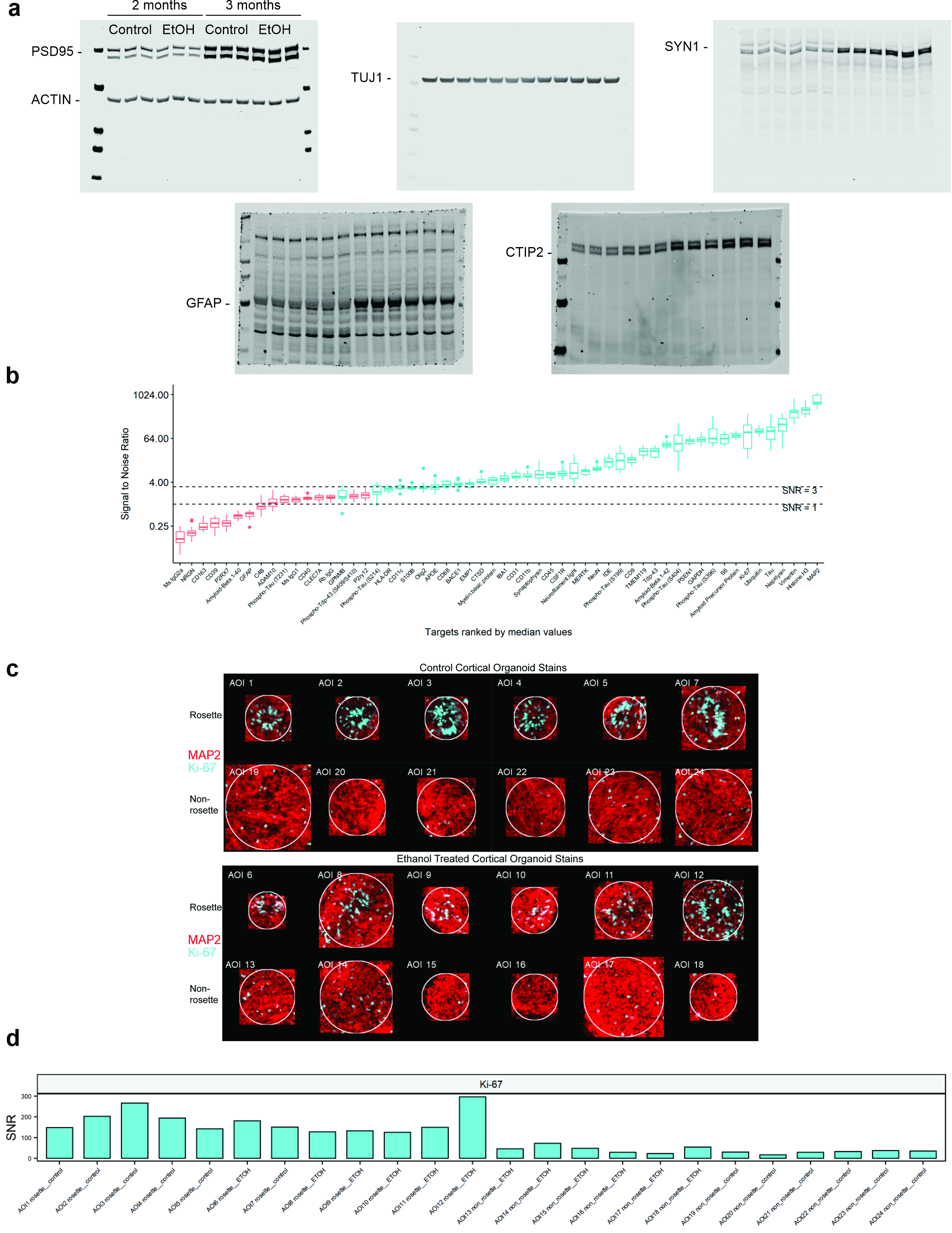

Supplement: Supplementary file 6 — Supplementary Figure 5 [file 41380_2022_1862_MOESM6_ESM.tif]
